# Evaluation of self-care with feet among patients with diabetes mellitus

**DOI:** 10.1590/1677-5449.210011

**Published:** 2022-01-31

**Authors:** Lorrany Junia Lopes de Lima, Matheus Rodrigues Lopes, Carlos Alberto de Lima Botelho, Roberta Stofeles Cecon

**Affiliations:** 1 Universidade Federal do Vale do São Francisco – UNIVASF, Campus Paulo Afonso, Paulo Afonso, BA, Brasil.

**Keywords:** diabetes mellitus, diabetic foot, self-care, health education

## Abstract

**Background:**

The diabetic foot is a complication of diabetes mellitus (DM) and is the most common cause of lower limb amputation.

**Objectives:**

To assess foot self-care practices by sex and educational level in DM patients from the Northeast of Brazil, state of Bahia.

**Methods:**

This was a quantitative, cross-sectional, observational, analytical study with 88 DM patients seen at routine consultations from February to March of 2020. Data were collected using questionnaires on socioeconomic data and self-care of feet (knowledge about the diabetic foot, habits related to care/inspection of feet, and visits to the Healthcare Center when changes to foot health are detected).

**Results:**

58% of the sample did not know the term “diabetic foot”, but a majority did perform minimum adequate foot care practices, such as inspecting feet (60.2%), moisturizing feet (65.9%), avoiding walking barefoot (81.8%), and trimming toenails (92%), although 90.9% did not wear footwear considered appropriate. There was a relationship between lower educational level and worse performance in questions relating to walking barefoot, moisturizing feet, trimming toenails, wearing appropriate footwear, and identifying mycoses (p < 0.05), but there was no association between performing self-care activities and sex.

**Conclusions:**

**Interviewed patients** with DM did not perform all foot self-care activities and did not know what the term “diabetic foot” means. There was an association between lower educational level and reduced capacity to perform these activities, which suggests that health literacy is important to improve self-care of feet, contributing to reduce complications and foot amputations.

## INTRODUCTION

Non-transmissible chronic diseases (NTCD) are considered one of the greatest public health challenges worldwide, responsible for around 38 million deaths annually, i.e., 70% of deaths globally. In Brazil, NTCD were the cause of 56.9% of deaths registered in 2017^1^^,^^2^.

Diabetes mellitus (DM) is one of the most prevalent NTCD and is characterized by a persistent hyperglycemic state^3^^,^^4^. The two primary categories of the disease are DM type 1, which is linked to autoimmune factors, and DM type 2, correlated with peripheral insulin resistance and progressive insufficiency of pancreatic beta cells^5^.

Failure of adequate glycemic control causes many acute complications (hypoglycemia, hyperosmolar hyperglycemic state, and diabetic ketoacidosis) and chronic conditions (diabetic foot, retinopathy, heart disease, nephropathy, neuropathies, cerebrovascular disease, and peripheral vascular disease)^6^, among which the diabetic foot is one of the most common complications of DM and an important cause of morbidity that can be averted with health education measures to teach and encourage self-care of the feet^7^.

The diabetic foot is defined as infections, ulcerations, and/or destruction of deep tissues, associated with neurological abnormalities, varying degrees of peripheral vascular disease in the lower limbs, and deficient glycemic control^7^^,^^8^. The risk of patients with DM developing foot ulcers is around 25%, with mortality after ulceration in the range of 43 to 55% within 5 years of the original event^7^^,^^8^.

The diabetic foot presents a mutilated appearance and is the greatest cause of non-traumatic lower limb amputations, which are primarily associated with osteomyelitis and wound infections^8^. In Brazil, the Brazilian National Health Service (SUS - *Sistema Único de Saúde*) conducted 102,056 amputation surgeries from 2011 to 2016, 94% of which were lower limb amputations, while 70% were in patients with DM and 85% were preceded by unprevented ulcers^9^^,^^10^. The diabetic foot has drastic consequences for the patient, because it causes reduced quality of life^11^^-^14.

It was found that the diabetic foot is associated with healthcare system expenditure, basically related with hospitalization and amputation^15^. Foot ulcers are responsible for 15% of the total estimated costs of DM in developed countries and 40% of expenditure in developing countries^16^; however, diabetic foot complications are highly avoidable with regular self-care practices stimulated by health education that raises patients’ awareness of the complications caused by diabetic feet^17^.

Habitual self-care practices are of fundamental importance to prevention and/or early detection of injuries that can result in ulceration, and constitute the most economic method of managing health^8^. Caring for the diabetic foot is one of the pillars of DM self-care^18^, which is the reason why educational training on self-care of feet is necessary, primarily within the primary healthcare (PHC) setting^8^. Therefore, the objective of this study was to assess the practice of foot self-care measures among patients with DM, by sex and educational level.

## METHODS

This was a quantitative, cross-sectional, observational, analytical study conducted in the municipal district of Paulo Afonso, in the Brazilian state of Bahia, with patients with DM cared for on the SUS, selected by non-probabilistic random sampling, i.e. by convenience. Data were collected weekly in the waiting room from patients attending routine medical consultations at the municipal district’s Medical Specialties Center from February to March of 2020.

The town of Paulo Afonso is located in the Northeast of Brazil and has an estimated population of 118,516 inhabitants, the mean monthly wage of its formally employed workers is 2.2 times the minimum wage, and 43.6% of town’s population have a per capita income of 0.5 times the minimum wage or less. The municipal district has a 96.4% rate of education from 6 to 14 years of age, and its public healthcare network offers all levels of healthcare^19^.

Participants were selected for the study according to the following inclusion criteria: age greater than or equal to 18 years, diagnosed with DM, and registered with the municipal district’s primary healthcare service. Exclusion criteria were disorders affecting the neurological system or hearing deficiencies that could prevent them from answering the questionnaires.

Patients were invited to participate while waiting for their medical consultations, prioritizing those at the back of the queue, to optimize use of time and encourage participation. Losses and refusals were not recorded.

The outcome analyzed was knowledge of and/or practice of foot self-care measures. The independent variables were sex (male; female); educational level (primary education; secondary education; higher education, incomplete or completed); and access to private healthcare services (yes; no).

Interviews were conducted by one of the study authors, who had been trained in advance, and took place in a secluded area in the waiting room, to maintain the patients’ privacy. Each interview lasted 30 minutes. After administration of the questionnaires, interviewees were given healthcare advice and pamphlets on self-care of the diabetic foot.

Interviews encompassed administration of two different questionnaires to assess the following variables:

- Sociodemographic data (prepared by the study authors); and
- Self-care of the diabetic foot (prepared by the study authors).

The questionnaire on self-care of the diabetic foot assesses self-care activities with questions about knowledge about what the diabetic foot is, habits of inspecting the feet and caring for the feet, in addition to visiting a primary care health center (PHCC) if any changes affecting the health of the feet are detected. The data collected were analyzed to identify which habits were least often practiced by the DM patients.

A database was constructed using Excel software. Data were analyzed using descriptive statistics and chi-square tests (to test the hypothesis of relationships between practicing foot self-care measures and patients’ sex or educational level), using SPSS for Windows, version 21.0. A 5% significance level was adopted.

The Municipal Health Department granted permission to conduct the study, which was approved by the Research Ethics Committee (CAAE: 21944919.6.0000.5196, under decision number 3.830.587, on February 10, 2020). All of the participants were invited to sign a free and informed consent form after the study and its objectives had been explained to them. Only researchers directly involved with the interviews had access to the study data and they signed a data use form committing themselves to maintain patient confidentiality and privacy.

## RESULTS

A total of 88 DM patients were interviewed. Mean age was 57.6±13.2 years. There was a predominance of female patients (77.3%). A majority of the participants had spent from 8 to 11 years in education and around 15% them stated that they were illiterate. Approximately 70% of the patients reported that they only had access to the public healthcare system (Table 1).

**Table 1:** Socioeconomic profile of the diabetes mellitus patients.

**Variables**	**n (%)**
Sex	
Female	68 (77.3)
Male	20 (22.7)
Educational level	
Illiterate	13 (14.7)
Primary education, incomplete	17 (19.3)
Primary education, completed	29 (33.0)
Secondary education, completed	26 (29.5)
Higher education, completed	3 (3.4)
Access to private healthcare	
Yes	27 (30.7)
No	61 (69.3)

n = number of participants; % = percentage of sample.

The majority of the DM patients stated that they were “homemakers”, retired, or unemployed, accounting for 31.8%, 33%, and 9.1% of the sample, respectively. The median time since diagnosis of DM was 8 years, ranging from 1 month to 30 years.

Median monthly family income was R$ 1,045.00 and the number of family members dependent on this income ranged from one to eight people, with a median of three.

The majority of the interviewees did not know about the diabetes complication the diabetic foot. The most prevalent care activities were avoiding walking barefoot and trimming toenails. The results for care related to daily inspection and hydration of feet showed that, although these measures were performed by the majority of the patients, there is still a need for the healthcare team that works at the PHC to emphasize their importance. It was observed that 90.9% of the DM patients did not wear appropriate footwear. Additionally, drying between the toes after washing feet was another self-care habit that was rarely performed. Around 49% of the interviewees reported that they did not visit the PHCC if they identified foot injuries or other changes before employing home remedies (Table 2).

**Table 2:** Assessment of knowledge about foot self-care measures among diabetes mellitus patients.

**Questions**	**Responses**	
	**Yes n (%)**	**No n (%)**
1. Do you know what the diabetic foot is?	37 (42)	51 (58)
2. Do you habitually inspect your feet daily?	53 (60.2)	35 (39.8)
3. Do you habitually walk barefoot?	16 (18.2)	72 (81.8)
4.Do you habitually moisturize your feet with cream or oil?	58 (65.9)	30 (34.1)
5. Do you care for your feet by trimming your toenails?	81 (92)	7 (8)
6. Do you wear shoes that are appropriate for people with diabetes?	8 (9.0)	80 (91.0)
7. Have you ever noticed a fungal infection between your toes?	27 (30.7)	61 (69.3)
8. Have you ever noticed that you have calluses on your feet?	39 (44.3)	49 (55.7)
9. Have you ever noticed that you have cracks on your feet?	51 (58)	37 (42)
10. Do you habitually dry the spaces between your toes after washing your feet?	48 (54.5)	40 (45.5)
11. Do you visit the PHCC when you notice damage and/or abnormalities before trying home treatments?	45 (51.1)	43 (48.9)

PHCC = Primary care health center; n = number of participants; % = percentage of sample.

When care procedures were analyzed by years in education, 84.6% and 64.7% of diabetes patients who self-identified as illiterate or reported not having completed primary education, respectively, were unaware of the diabetic foot. According to the answers to the question about inspecting feet, 76.9% of illiterate patients and 41.2% of those who had not completed primary education did not conduct foot inspections. When asked if they visit the PHCC if they detect foot lesions, 58.3% of illiterate participants replied “no”. There were relationships between lower educational level and worse results for questions on walking barefoot, moisturizing feet, trimming toenails, wearing appropriate footwear, and identifying mycoses (p < 0.05) (Table 3).

**Table 3:** Analysis of foot care activities by people with diabetes mellitus, by educational level.

**Questions**	**Responses**										**p** *
	**Illiterate**		**Primary education, incomplete**		**Primary education, completed**		**Secondary education, completed**		**Higher education, completed**		
	**Yes n (%)**	**No n (%)**	**Yes n (%)**	**No n (%)**	**Yes n (%)**	**No n (%)**	**Yes n (%)**	**No n (%)**	**Yes n (%)**	**No n (%)**	
1. Do you know what the diabetic foot is?	2 (2.3)	11 (12.5)	6 (6.8)	11 (12.5)	12 (13.6)	17 (19.3)	15 (17.1)	11 (12.5)	2 (2.3)	1 (1.1)	0.14
2. Do you habitually inspect your feet daily?	3 (3.4)	10 (11.4)	10 (11.4)	7 (7.9)	21 (23.9)	8 (9.0)	16 (18.2)	10 (11.4)	3 (3.4)	0 (0.0)	0.06
3. Do you habitually walk barefoot?	1 (1.1)	12 (13.6)	2 (2.3)	15 (17.0)	7 (7.9)	22 (25.0)	4 (4.6)	22 (25.0)	2 (2.3)	1 (1.1)	<0.01
4.Do you habitually moisturize your feet with cream or oil?	10 (11.4)	3 (3.4)	7 (7.9)	10 (11.4)	23 (26.1)	6 (6.8)	17 (19.3)	9 (10.2)	1 (1.1)	2 (2.3)	<0.01
5. Do you care for your feet by trimming your toenails?	13 (14.8)	0 (0.0)	16 (18.2)	1 (1.1)	24 (27.3)	5 (5.7)	25 (28.4)	1 (1.1)	3 (3.4)	0 (0.0)	<0.01
6. Do you wear shoes that are appropriate for people with diabetes?	0 (0.0)	13 (14.8)	4 (4.6)	13 (14.8)	2 (2.3)	27 (30.6)	1 (1.1)	25 (28.4)	1 (1.1)	2 (2.3)	<0.01
7. Have you ever noticed a fungal infection between your toes?	2 (2.3)	11 (12.5)	8 (9.0)	9 (10.2)	10 (11.4)	19 (21.6)	5 (5.7)	21 (23.9)	2 (2.3)	1 (1.1)	<0.01
8. Have you ever noticed that you have calluses on your feet?	5 (5.7)	8 (9.0)	7 (7.9)	10 (11.4)	14 (15.9)	15 (17.1)	11 (12.5)	15 (17.0)	2 (2.3)	1 (1.1)	0.29
9. Have you ever noticed that you have cracks on your feet?	8 (9.0)	5 (5.7)	11 (12.5)	6 (6.8)	15 (17.1)	14 (15.9)	16 (18.2)	10 (11.4)	1 (1.1)	2 (2.3)	0.14
10. Do you habitually dry the spaces between your toes after washing your feet?	8 (9.0)	5 (5.7)	10 (11.4)	7 (7.9)	16 (18.2)	13 (14.8)	13 (14.8)	13 (14.8)	1 (1.1)	2 (2.3)	0.39
11. Do you visit the PHCC when you notice damage and/or abnormalities before trying home treatments?	5 (5.7)	8 (9.0)	10 (11.4)	7 (7.9)	16 (18.2)	13 (14.8)	13 (14.8)	13 (14.8)	1 (1.1)	2 (2.3)	0.83

* Chi-square test (*p*-value). Significance level do test: <0.05;

PHCC = Primary care health center; n = number of participants; % = percentage of sample.

With relation to sex, 10.2% of the men and 31.8% of the women stated they knew about the diabetic foot. The percentages of some responses to questions related to self-care activities differed between women and men, with greater differences for walking barefoot, moisturizing feet, trimming toenails, and wearing footwear appropriate for DM patients, but without statistically significant differences between the two groups (Table 4).

**Table 4:** Analysis of foot care activities by people with diabetes mellitus, by sex.

**Questions**	**Responses**				
	**Yes**		**No**		**p** *
	**F n (%)**	**M n (%)**	**F n (%)**	**M n (%)**	
1. Do you know what the diabetic foot is?	28 (31.8)	9 (10.2)	40 (45.5)	11 (12.5)	0.77
2. Do you habitually inspect your feet daily?	41 (46.6)	12 (13.6)	27 (30.7)	8 (9.1)	0.98
3. Do you habitually walk barefoot?	14 (15.9)	2 (2.3)	54 (61.3)	18 (20.5)	0.28
4.Do you habitually moisturize your feet with cream or oil?	48 (54.5)	10 (11.4)	20 (22.7)	10 (11.4)	0.09
5. Do you care for your feet by trimming your toenails?	63 (71.5)	18 (20.5)	5 (5.7)	2 (2.3)	0.70
6. Do you wear shoes that are appropriate for people with diabetes?	4 (4.5)	4 (4.5)	64 (72.8)	16 (18.2)	0.05
7. Have you ever noticed a fungal infection between your toes?	19 (21.6)	8 (9.1)	49 (55.7)	12 (13.6)	0.30
8. Have you ever noticed that you have calluses on your feet?	29 (32.9)	10 (11.4)	39 (44.3)	10 (11.4)	0.56
9. Have you ever noticed that you have cracks on your feet?	40 (45.5)	11 (12.5)	28 (31.8)	9 (10.2)	0.76
10. Do you habitually dry the spaces between your toes after washing your feet?	39 (44.3)	9 (10.2)	29 (32.9)	11 (12.5)	0.33
11. Do you visit the PHCC when you notice damage and/or abnormalities before trying home treatments?	36 (40.9)	9 (10.2)	32 (36.4)	11 (12.5)	0.53

* Chi-square test (*p*-value). Significance level of the test: <0.05;

PHCC = Primary care health center; F = female; M = male; n = number of participants; % = percentage of sample.

## DISCUSSION

Mean age was 57.6 years, which is a result corroborated by other scientific studies that have reported evidence of a proportional association between age and emergence of NTCDs^20^^,^^21^. There was a higher number of female participants, since women are more likely to be concerned about disease and seek health services more frequently than men^22^.

There was a higher prevalence of individuals with lower educational levels. It has been reported that people with lower educational level are more likely to develop NTCDs, because of socioeconomic disadvantages, greater vulnerability and, consequently, reduced access to health services^23^^,^^24^. The low-income population is more affected by NTCDs, because it is subject to risk factors and has less access to measures for health promotion and prevention of disorders offered by health services^25^. Approximately 82% of the interviewees in this study had incomes of two times the minimum wage or less, which is similar to other studies conducted with DM patients^21^^,^^26^.

It was observed that almost 70% of the interviewees did not have access to the private healthcare system. People who exclusively use the SUS have a 60% greater likelihood of facing barriers to obtaining medical attention compared to patients with access to private healthcare^27^.

In this study, more than half of the patients stated that they did not know the term “diabetic foot”, which is a factor that could increase the incidence of this complication, since studies have shown evidence of a correlation between ignorance of the subject and increased incidence of the condition^28^. Health education is a fundamental axis in non-pharmaceutical treatment of DM, because it provides people with the capacity to self-manage their disease^29^. Thus, educational strategies enable the patient to achieve autonomy and develop the skills to deal with DM, resulting in adequate glycemic control and lower risk of complications^30^^,^^31^.

Patients who receive advice and instruction from health professionals and are encouraged to care for their diabetic feet acquire better self-care habits. Therefore, educational programs based on clinical practice guidelines should be encouraged and put into practice, in both PHCC and hospital settings^16^. These educational activities can be implemented in the form of creative and attractive measures, using verbal language appropriate to the patient, videos, animations, descriptive images, and group discussions^32^^,^^33^.

Additionally, the educational activities should be based on dialog, to encourage active participation of patients, and should be contextualized with simulations to train them to perform self-care adequately^33^. These approaches are relevant to reduce complications, since studies report evidence showing that 49 to 85% of ulcers can be avoided with health education that results in patient adherence to the treatment plan and, consequently, to self-care^16^^,^^22^.

The majority of interviewees claimed that they were in the habit of inspecting their own feet, which is an important measure for detecting early signs of ulceration, such as a presence of edema, erythema, dry skin, discoloration, and lesions such as calluses, wounds, punctures, and cuts^32^^,^^34^.

More than 65% of the DM patients interviewed moisturized their feet, which is a result that is compatible with other studies^15^^,^^34^. Dry skin is common in DM because of the sudomotor disorders caused by autonomic neuropathy. Moisturizers should therefore be applied three times a day with the objective of avoiding dry skin, which constitutes a medium susceptible to development of cracks and calluses^35^. Many of the patients interviewed stated that they had had cracked skin or calluses. These are pre-ulcerous lesions that facilitate entry of microorganisms and are precursors of infections and ulcerations^10^^,^^35^. Mycoses also constitute ports of entry for the emergence of infectious, particularly those of a polymicrobial nature, and presence of these problems was reported by around 30% of the patients assessed^34^^,^^35^. One measure for averting mycoses is to dry the spaces between the toes after washing feet^34^, a practice that 54.5% of the patients performed.

Almost 82% of the interviewees reported that they do not walk barefoot, a result that is similar to those of other studies^36^, and an essential self-care practice to avoid imperceptible external traumas and elevated mechanical stresses in DM patients. With relation to trimming toenails, 92% of the individuals assessed stated they did trim their toenails, but were not asked about the way they cut them, which should be done straight, filing the corners of the nails round, to prevent development of lesions and ingrowing toenails^33^.

Approximately 91% of the patients did not wear footwear appropriate for DM, which has also been observed in other studies, in which more than 90% of people with diabetes also had the same habit. The ideal footwear should not have internal stitching, should be soft, adjustable with laces or velcro, should offer total protection for the toes, should have a sole of 3 centimeters or less, should protect the feet from mechanical traumas, should be the correct size, and should distribute areas of pressure^10^^,^^15^.

Wearing adequate footwear should be part of the guidance provided to patients by healthcare professionals, because it reduces the risks of development of the first onset of ulcers and also reduces recurrence of ulceration in people who have abnormal plantar pressure because of healed ulcers^13^^,^^32^. Not wearing therapeutic shoes may be correlated with the high cost of this type of footwear, which may make them impossible to buy for many patients because of their low incomes. Another issue is their esthetics, since many people consider them to be ugly and prefer standard shoes, which can cause injuries^22^.

It was found that when they had foot lesions 48.9% of the interviewees did not visit the PHCC before performing home treatments, which is incompatible with correct self-care, since patients should be instructed to seek a healthcare professional when they detect changes and/or lesions^10^ and before performing home treatments that can lead to infections and ulcerations of the foot. Examples of incorrect methods are heating the feet with an iron or hot water bottle, or using inappropriate tools to cut calluses and treat cracks^8^.

The educational level of DM patients has a positive influence on their capacity to perform self-care of their feet^37^. It is expected that people with higher educational levels will have better comprehension of literature on the disease and its treatment and will also understand information provided by health professionals better and, thus, adopt adequate preventative behaviors^8^^,^^16^^,^^37^. This coincides with some of the self-care practices assessed, since 75% of illiterate participants did not inspect their feet daily and 58.3% of them did not visit the PHCC if they found lesions or changes to their feet. In the present study there were statistical differences in the answers to questions on walking barefoot, moisturizing feet, trimming toenails, wearing appropriate footwear, and identifying mycoses, revealing associations between these self-care practices and low educational level in DM patients. Health professionals should develop tailored educational interventions to help this population, with guidance delivered in a simple and objective manner, using language compatible with their educational level so that they transfer knowledge and encourage self-care^8^^,^^37^.

In our study, there were no statistical differences in responses on self-care according to the sex of respondents. It was observed that around 54.5% of the women were in the habit of moisturizing their feet, compared to 11.4% of the men. Other practices that were performed by a higher proportion of the females were taking care to dry feet after washing, and trimming toenails correctly, while a higher percentage of the men were in the habit of wearing adequate footwear for DM, which are results that are compatible with those of a study conducted in the South of Brazil^36^. The higher frequency of wearing appropriate footwear by men is related to the wide variety of footwear worn by the female population, including shoes with high heels, more openings, and greater exposure of the toes^38^. It is therefore important that health services take sex differences into consideration when designing educational interventions and more effective care plans^38^.

Diabetic foot ulcers are the most prevalent long term complications and around 20% of patients living with DM are at high risk of developing this complication in their feet because of the presence of this neuropathy^12^^,^^39^. Existence of ulceration is one of the principal causes of hospitalizations and is difficult to treat, since around 40% of ulcers do not heal in response to specific measures within the first 6 months^12^.

Diabetic foot complications are therefore correlated with low productivity, individual healthcare costs, difficulties with performing physical exercises, and problems related to anxiety, depression, and stress, in addition to problems that intensify feelings of social isolation. These conditions contribute to reduced quality of life among patients with DM^12^.

It is important to point out the limitations of this study. The total number of patients with DM assessed in the study may have been a limiting factor since it did not attain the sample size calculated (considering a 7.4% prevalence of physician-diagnosed DM in the VIGITEL 2019 study, the population of Paulo Afonso of 118,516 inhabitants, an acceptable margin of error of 5%, and a 95% significance level, which resulted in a sample size of 105 individuals). Nevertheless, this sample still allows for inferences and extrapolation of the results, since it is representative of the population with DM. Additionally, the number of people who refused to participate in the study and were excluded was not quantified; but these limitations do not impact the importance of the study, since the subject is of fundamental importance to organization of educational interventions in health services.

## CONCLUSIONS

It was found that interviewed patients with DM did not perform all of the recommended foot self-care procedures, such as daily inspection, moisturizing, toenail trimming, drying the spaces between toes, and wearing appropriate footwear, in addition to not knowing what the tem “diabetic foot” means. There was an association between lower educational level and lower capacity to perform foot self-care procedures, but there was no relationship between these self-care activities and sex.

In this context, it is inferred that health education and health literacy, provided and or reinforced by the health professionals at the PHC in a manner tailored to each patient profile are important to improve self-care of feet and can contribute to reduce complications, hospitalizations, and amputations among patients with DM.
