# Supplementary material for: Evaluation of self-care with feet among patients with diabetes mellitus
Source: J Vasc Bras. 2022 Jan 31;21:e20210011. doi: 10.1590/1677-5449.210011 (PMC8862594; doi:10.1590/1677-5449.210011)
Supplement: Translation

# Translation: Avaliação do autocuidado com os pés entre pacientes portadores de diabetes melito

**Authors:** Lorrany Junia Lopes de Lima, Matheus Rodrigues Lopes, Carlos Alberto de Lima Botelho, Roberta Stofeles Cecon

**Affiliations:** 1 Universidade Federal do Vale do São Francisco – UNIVASF, Campus Paulo Afonso, Paulo Afonso, BA, Brasil.

**Keywords:** diabetes melito, pé diabético, autocuidado, educação em saúde

## Abstract

**Contexto:**

O pé diabético é uma complicação do diabetes melito (DM), sendo a maior causa de amputação dos membros inferiores.

**Objetivos:**

Avaliar a prática de medidas de autocuidado com os pés, segundo sexo e escolaridade, em pacientes portadores de DM na região nordeste no estado da Bahia.

**Métodos:**

Estudo quantitativo, observacional, analítico, transversal, realizado com 88 pacientes portadores de DM, em consulta de rotina, de fevereiro a março de 2020. A coleta de dados foi executada através da aplicação de questionários socioeconômico e do autocuidado com os pés (conhecimento sobre pé diabético, hábitos de cuidado/inspeção dos pés e procura pela Unidade de Saúde na presença de alterações com a saúde dos pés).

**Resultados:**

Do total, 58% dos indivíduos desconhecia o termo “pé diabético”, porém possuíam cuidados mínimos adequados com os pés, como inspecioná-los (60,2%), hidratá-los (65,9%), não andar descalço (81,8%) e cortar as unhas (92%), apesar de 90,9% não utilizar sapatos considerados adequados. Houve relação entre menor nível de escolaridade e pior desempenho nas questões referentes a andar descalço, hidratar os pés, cortar as unhas, usar calçados adequados e identificar micoses (p < 0,05), porém não houve associação da realização das medidas de autocuidado e sexo.

**Conclusão:**

Os portadores de DM entrevistados não realizaram todas as medidas de autocuidado com os pés e desconheciam o termo “pé diabético”. Houve associação entre menor escolaridade e menor capacidade de realização dessas medidas, o que sugere que o letramento em saúde seria importante para melhoria desse autocuidado, contribuindo para diminuição de complicações e amputações dos pés.

## INTRODUÇÃO

As doenças crônicas não transmissíveis (DCNT) são consideradas um dos maiores desafios da saúde pública mundial, responsáveis por cerca de 38 milhões de óbitos anualmente, ou seja, 70% das mortes no mundo. No Brasil, 56,9% das mortes ocorridas no ano de 2017 foram em razão das DCNT^1^^,^^2^.

O diabetes melito (DM), caracterizado por quadro de hiperglicemia persistente, é uma das DCNT mais prevalentes^3^^,^^4^. As duas principais classificações da doença correspondem ao DM tipo 1, associado a fatores autoimunes, e o DM tipo 2, correlacionado com a resistência periférica à insulina e com a insuficiência gradual das células beta pancreáticas^5^.

A ausência de controle glicêmico adequado ocasiona diversas complicações de caráter agudo (hipoglicemia, estado hiperglicêmico hiperosmolar e cetoacidose diabética) e também de condição crônica (pé diabético, retinopatia, cardiopatia, nefropatia, neuropatias, doença cerebrovascular e vascular periférica)^6^, sendo o pé diabético uma das principais complicações do DM e uma importante causa de morbidade que pode ser evitada com medidas de educação em saúde que estimulem o autocuidado com os pés^7^.

O pé diabético é definido como infecções, ulcerações e/ou destruição dos tecidos profundos, associados a anormalidades neurológicas, vários graus de doença vascular periférica nos membros inferiores e controle glicêmico deficiente^7^^,^^8^. O risco do paciente portador de DM de desenvolver úlcera nos pés gira em torno de 25%, com taxa de mortalidade após quadro de ulceração de 43 a 55% após 5 anos do acontecimento^7^^,^^8^.

O pé diabético possui aspecto mutilador e constitui a maior causa de amputação não traumática em membros inferiores, principalmente associado com osteomielite e infecção da ferida^8^. No Brasil, entre 2011 e 2016, ocorreram 102.056 cirurgias de amputação pelo Sistema Único de Saúde (SUS), sendo 94% do membro inferior, 70% em pacientes com DM e 85% antecedidas de úlcera não evitada^9^^,^^10^. O pé diabético gera consequências drásticas para o paciente, pois ocasiona baixa qualidade de vida^11^^-^14.

Verifica-se que o pé diabético associa-se com gastos no sistema de saúde, relacionados essencialmente com internação e amputação^15^. A presença de úlcera nos pés representa 15% do custo total estimado de DM nos países desenvolvidos e 40% dos gastos nos países em desenvolvimento^16^; entretanto, as complicações do pé diabético caracterizam-se por serem altamente evitáveis com a prática regular do autocuidado proveniente de uma educação em saúde que conscientiza os indivíduos acerca das complicações do pé diabético^17^.

A prática habitual do autocuidado é fundamental para prevenir e/ou detectar precocemente lesões que podem levar a ulceração, sendo a forma mais econômica para gerar saúde^8^. O cuidado com o pé diabético é um dos pilares do autocuidado no DM^18^, e, por isso, são necessários treinamentos educativos de autocuidado com os pés, principalmente na atenção primária à saúde (APS)^8^. Portanto, este estudo objetiva avaliar a prática de medidas de autocuidado com os pés, segundo sexo e escolaridade, em portadores de DM.

## MÉTODOS

Tratou-se de estudo quantitativo, observacional, analítico e transversal realizado no município de Paulo Afonso, no estado da Bahia, com pacientes portadores de DM atendidos pelo SUS e selecionados por amostragem a esmo, não probabilística, ou seja, por conveniência. A coleta de dados foi tipo sala de espera, realizada com pacientes em consulta médica de rotina, desenvolvida semanalmente no Centro de Especialidades Médicas do município no período de fevereiro a março de 2020.

A cidade de Paulo Afonso é localizada na região nordeste do Brasil e conta com um número estimado de 118.516 habitantes, o salário médio mensal dos trabalhadores formais é de 2,2 salários-mínimos, e 43,6% da população tem rendimento per capita de até 0,5 salário-mínimo. O município conta com taxa de escolarização de 6 a 14 anos de idade de 96,4% e rede de serviços de saúde estruturada com todos os níveis de atenção^19^.

Os indivíduos foram selecionados para a pesquisa seguindo-se os critérios de inclusão de ter idade igual ou superior a 18 anos, ser portador de DM e ser usuário cadastrado da atenção básica do município. Os critérios de exclusão foram a presença de distúrbios do sistema neurológico ou de audição deficientes que impedissem os indivíduos de responderem aos questionários.

Os pacientes foram convocados durante o tempo de espera antes da consulta médica, priorizando os últimos indivíduos da fila de espera, para otimizar o tempo e incentivar a adesão à pesquisa. Não foi feito o registro de perdas e recusas.

O desfecho consistiu no conhecimento/realização das medidas de autocuidado com os pés. As variáveis independentes foram sexo (masculino; feminino); escolaridade (ensino fundamental; ensino médio; ensino superior completo ou incompleto); e acesso ao serviço privado de saúde (sim; não).

As entrevistas foram realizadas por uma das pesquisadoras do estudo, previamente treinada, e ocorreram em lugar reservado, na sala de espera, para garantir sigilo aos pacientes. A duração foi de 30 minutos para cada entrevistado. Após a aplicação dos questionários, foram realizadas orientações em saúde e entrega de panfletos sobre o autocuidado com o pé diabético.

Foram realizadas entrevistas com a aplicação de questionários distintos para avaliar as seguintes variáveis:

- Sociodemográficas (elaborado pelos pesquisadores do estudo); e
- Autocuidado com o pé diabético (elaborado pelos pesquisadores do estudo).

O questionário para o autocuidado com o pé diabético avalia medidas de autocuidado, com perguntas relacionadas ao conhecimento sobre o que é o pé diabético, o hábito de inspecionar os pés e cuidado com os mesmos, além da procura pela Unidade Básica de Saúde (UBS) na presença de alguma alteração com a saúde dos pés. Por meio da análise dos dados obtidos, foi possível avaliar os hábitos menos realizados pelos portadores de DM.

O banco de dados foi elaborado no *software* Excel. Os dados foram analisados por meio de estatística descritiva e teste qui-quadrado (testar a hipótese da relação entre a realização de medidas de cuidados com os pés com o sexo e a escolaridade dos pacientes), com o auxílio do *software* SPSS for Windows, versão 21.0. Foi adotado nível de significância de 5%.

O estudo recebeu a anuência da Secretaria Municipal de Saúde e aprovação do Comitê de Ética em Pesquisa (CAAE: 21944919.6.0000.5196, sob parecer número 3.830.587 de 10 de fevereiro de 2020). Todos os participantes foram convidados a assinar o termo de consentimento livre e esclarecido após explicação sobre a pesquisa e os objetivos do estudo. Apenas os pesquisadores diretamente envolvidos com as entrevistas tiveram acesso aos dados, e esses pesquisadores assinaram termo assumindo o compromisso de manter confidencialidade e sigilo sobre todas as informações.

## RESULTADOS

Foram entrevistados 88 pacientes portadores de DM, com idade média de 57,6±13,2 anos. Verificou-se prevalência do sexo feminino (77,3%). A maior parte dos indivíduos apresentou de 8 a 11 anos de estudo, e cerca de 15% dos portadores de DM relataram ser analfabetos. Aproximadamente 70% dos pacientes relataram ter acesso somente ao serviço público de saúde (Tabela 1).

**Tabela 1:** Perfil socioeconômico dos pacientes portadores de diabetes melito.

**Variáveis**	**n (%)**
Sexo	
Feminino	68 (77,3)
Masculino	20 (22,7)
Escolaridade	
Analfabeto	13 (14,7)
Ensino fundamental incompleto	17 (19,3)
Ensino fundamental completo	29 (33,0)
Ensino médio completo	26 (29,5)
Ensino superior completo	3 (3,4)
Acesso ao serviço privado de saúde	
Sim	27 (30,7)
Não	61 (69,3)

n = número amostral; % = porcentagem.

A maioria dos pacientes portadores de DM relatou ser “do lar”, aposentados e desempregados, com valores de 31,8%, 33% e 9,1%, respectivamente. O tempo de diagnóstico do DM apresentou mediana de 8 anos, mínimo de 1 mês e máximo de 30 anos.

A renda mensal das famílias apresentou mediana de R$ 1.045,00, e o número de membros, por famílias, que dependiam dessa renda variou entre um a oito indivíduos, com mediana de três membros.

A maioria dos entrevistados desconhecia a complicação do pé diabético. As atividades mais realizadas foram relacionadas ao cuidado de não andar descalço e o cuidado ao cortar as unhas dos pés. Os resultados dos cuidados relacionados à inspeção e hidratação dos pés diariamente mostra que, apesar de realizados pela maioria dos pacientes, ainda necessita de reforço por parte da equipe de saúde que atua na APS. Foi visto que 90,9% dos portadores de DM não utilizavam sapatos adequados. Além disso, secar os espaços interdigitais após a lavagem dos pés é um hábito de autocuidado ainda pouco realizado. Cerca de 49% dos entrevistados relataram que não procuravam pela UBS na presença de lesões e/ou alterações no pé antes de realizarem procedimentos caseiros (Tabela 2).

**Tabela 2:** Avaliação do conhecimento sobre medidas de autocuidado com o pé em portadores de diabetes melito.

**Questões**	**Respostas**	
	**Sim n (%)**	**Não n (%)**
1. Você sabe o que é o pé diabético?	37 (42)	51 (58)
2. Você possui o hábito de inspecionar os pés diariamente?	53 (60,2)	35 (39,8)
3. Você possui o hábito de andar descalço?	16 (18,2)	72 (81,8)
4.Você possui o hábito de hidratar os pés com cremes ou óleos?	58 (65,9)	30 (34,1)
5. Você possui cuidados ao cortar as unhas dos pés?	81 (92)	7 (8)
6. Você usa sapatos adequados para o diabetes?	8 (9,0)	80 (91,0)
7. Você já notou se há presença de micose interdigital nos pés?	27 (30,7)	61 (69,3)
8. Você já notou se há presença de calos nos pés?	39 (44,3)	49 (55,7)
9. Você já notou se há presença de rachaduras nos pés?	51 (58)	37 (42)
10. Você possui o hábito de secar os espaços interdigitais após a lavagem dos pés?	48 (54,5)	40 (45,5)
11. Você procura pela UBS na presença de lesões e/ou alterações antes de realizar procedimentos caseiros?	45 (51,1)	43 (48,9)

UBS = Unidade Básica de Saúde; n = número amostral; % = porcentagem.

Quando as medidas de cuidado foram analisadas pelos anos de estudo dos portadores de diabetes entre os pacientes declarados analfabetos e com ensino fundamental incompleto, 84,6% e 64,7%, respectivamente, desconheciam o pé diabético. De acordo com a questão acerca do hábito de inspecionar os pés, 76,9% dos analfabetos e 41,2% dos pacientes com ensino fundamental incompleto não realizavam essa atividade. Quando questionados acerca de procurar a UBS diante da presença de lesões nos pés, 58,3% dos analfabetos responderam “não”. Constatou-se relação entre menor nível de escolaridade com pior desempenho nas questões referentes aos costumes de andar descalço, hidratar os pés, cortar as unhas, usar calçados adequados e identificar micoses (p < 0,05) (Tabela 3).

**Tabela 3:** Análise sobre a realização de medidas de cuidados com os pés em portadores de diabetes melito, segundo a escolaridade.

**Questões**	**Respostas**										**p** *
	**Analfabeto**		**Ensino fundamental incompleto**		**Ensino fundamental completo**		**Ensino médio completo**		**Ensino superior completo**		
	**Sim n (%)**	**Não n (%)**	**Sim n (%)**	**Não n (%)**	**Sim n (%)**	**Não n (%)**	**Sim n (%)**	**Não n (%)**	**Sim n (%)**	**Não n (%)**	
1. Você sabe o que é o pé diabético?	2 (2,3)	11 (12,5)	6 (6,8)	11 (12,5)	12 (13,6)	17 (19,3)	15 (17,1)	11 (12,5)	2 (2,3)	1 (1,1)	0,14
2. Você possui o hábito de inspecionar os pés diariamente?	3 (3,4)	10 (11,4)	10 (11,4)	7 (7,9)	21 (23,9)	8 (9,0)	16 (18,2)	10 (11,4)	3 (3,4)	0 (0,0)	0,06
3. Você possui o hábito de andar descalço?	1 (1,1)	12 (13,6)	2 (2,3)	15 (17,0)	7 (7,9)	22 (25,0)	4 (4,6)	22 (25,0)	2 (2,3)	1 (1,1)	<0,01
4. Você possui o hábito de hidratar os pés com cremes ou óleos?	10 (11,4)	3 (3,4)	7 (7,9)	10 (11,4)	23 (26,1)	6 (6,8)	17 (19,3)	9 (10,2)	1 (1,1)	2 (2,3)	<0,01
5. Você possui cuidados ao cortar as unhas dos pés?	13 (14,8)	0 (0,0)	16 (18,2)	1 (1,1)	24 (27,3)	5 (5,7)	25 (28,4)	1 (1,1)	3 (3,4)	0 (0,0)	<0,01
6. Você usa sapatos adequados para o diabetes?	0 (0,0)	13 (14,8)	4 (4,6)	13 (14,8)	2 (2,3)	27 (30,6)	1 (1,1)	25 (28,4)	1 (1,1)	2 (2,3)	<0,01
7. Você já notou se há presença de micose interdigital nos pés?	2 (2,3)	11 (12,5)	8 (9,0)	9 (10,2)	10 (11,4)	19 (21,6)	5 (5,7)	21 (23,9)	2 (2,3)	1 (1,1)	<0,01
8. Você já notou se há presença de calos nos pés?	5 (5,7)	8 (9,0)	7 (7,9)	10 (11,4)	14 (15,9)	15 (17,1)	11 (12,5)	15 (17,0)	2 (2,3)	1 (1,1)	0,29
9. Você já notou se há presença de rachaduras nos pés?	8 (9,0)	5 (5,7)	11 (12,5)	6 (6,8)	15 (17,1)	14 (15,9)	16 (18,2)	10 (11,4)	1 (1,1)	2 (2,3)	0,14
10. Você possui o hábito de secar os espaços interdigitais após a lavagem dos pés?	8 (9,0)	5 (5,7)	10 (11,4)	7 (7,9)	16 (18,2)	13 (14,8)	13 (14,8)	13 (14,8)	1 (1,1)	2 (2,3)	0,39
11. Você procura pela UBS na presença de lesões e/ou alterações antes de realizar procedimentos caseiros?	5 (5,7)	8 (9,0)	10 (11,4)	7 (7,9)	16 (18,2)	13 (14,8)	13 (14,8)	13 (14,8)	1 (1,1)	2 (2,3)	0,83

* Teste qui-quadrado (p-valor). Nível de significância do teste: <0,05;

UBS = Unidade Básica de Saúde; n = número amostral; % = porcentagem.

Em relação ao sexo, 10,2% dos homens e 31,8% das mulheres afirmaram conhecer acerca do pé diabético. Quanto às questões relacionadas às atividades de autocuidado, houve algumas diferenças nas porcentagens de respostas entre as mulheres e os homens, com destaque para o hábito de andar descalço, o costume de hidratar os pés, o cuidado ao cortar as unhas dos pés e o uso de sapatos adequados para o DM, porém, sem diferença estatística entre os grupos (Tabela 4).

**Tabela 4:** Análise sobre a realização de medidas de cuidados com os pés em portadores de diabetes melito, de acordo com o sexo.

**Questões**	**Respostas**				
	**Sim**		**Não**		**p** *
	**F n (%)**	**M n (%)**	**F n (%)**	**M n (%)**	
1. Você sabe o que é o pé diabético?	28 (31,8)	9 (10,2)	40 (45,5)	11 (12,5)	0,77
2. Você possui o hábito de inspecionar os pés diariamente?	41 (46,6)	12 (13,6)	27 (30,7)	8 (9,1)	0,98
3. Você possui o hábito de andar descalço?	14 (15,9)	2 (2,3)	54 (61,3)	18 (20,5)	0,28
4.Você possui o hábito de hidratar os pés com cremes ou óleos?	48 (54,5)	10 (11,4)	20 (22,7)	10 (11,4)	0,09
5. Você possui cuidados ao cortar as unhas dos pés?	63 (71,5)	18 (20,5)	5 (5,7)	2 (2,3)	0,70
6. Você usa sapatos adequados para o diabetes?	4 (4,5)	4 (4,5)	64 (72,8)	16 (18,2)	0,05
7. Você já notou se há presença de micose interdigital nos pés?	19 (21,6)	8 (9,1)	49 (55,7)	12 (13,6)	0,30
8. Você já notou se há presença de calos nos pés?	29 (32,9)	10 (11,4)	39 (44,3)	10 (11,4)	0,56
9. Você já notou se há presença de rachaduras nos pés?	40 (45,5)	11 (12,5)	28 (31,8)	9 (10,2)	0,76
10. Você possui o hábito de secar os espaços interdigitais após a lavagem dos pés?	39 (44,3)	9 (10,2)	29 (32,9)	11 (12,5)	0,33
11. Você procura pela UBS na presença de lesões e/ou alterações antes de realizar procedimentos caseiros?	36 (40,9)	9 (10,2)	32 (36,4)	11 (12,5)	0,53

* Teste qui-quadrado (p-valor). Nível de significância do teste: <0,05;

UBS = Unidade Básica de Saúde; F = feminino; M = masculino; n = número amostral; % = porcentagem.

## DISCUSSÃO

A média da idade foi de 57,6 anos, resultado corroborado por outros trabalhos científicos que evidenciam a associação proporcional entre a idade e o aparecimento das DCNT^20^^,^^21^. Observou-se maior número de participantes do sexo feminino, na medida em que mulheres possuem maior preocupação com a doença e procuram com mais frequência os serviços de saúde comparadas a homens^22^.

Houve maior prevalência de indivíduos com menor nível de escolaridade. É descrito que as pessoas com baixa escolaridade possuem maior probabilidade de desenvolver DCNT, devido às desvantagens socioeconômicas, maior vulnerabilidade e, consequentemente, menor acesso aos serviços de saúde^23^^,^^24^. A população de baixa renda é mais acometida por DCNT, pois está sujeita a fatores de risco e apresenta menor acesso às medidas de promoção de saúde e prevenção de agravos oferecidas pelos serviços de saúde^25^. Aproximadamente 82% dos entrevistados neste estudo recebiam até dois salários-mínimos mensais, fato similar a outros estudos realizados com pacientes portadores de DM^21^^,^^26^.

Observou-se que quase 70% dos entrevistados não tinham acesso ao sistema privado de saúde. Os usuários exclusivos do SUS têm chance 60% maior de terem empecilhos para obtenção de atendimento médico em relação aos pacientes com acesso ao serviço privado^27^.

Nesta pesquisa, mais da metade dos pacientes relataram não conhecer o termo “pé diabético”, fato que pode aumentar a incidência dessa complicação, pois estudos evidenciam a correlação do desconhecimento do assunto com o aumento da incidência da enfermidade^28^. A educação em saúde é o eixo fundamental do tratamento não medicamentoso do DM, pois proporciona a capacitação dos indivíduos para realizar o autogerenciamento da sua doença^29^. Assim, as estratégias educativas permitem que o indivíduo possua autonomia e desenvolva habilidades para lidar com o DM, o que resulta em controle glicêmico adequado e menores riscos de complicações^30^^,^^31^.

Pacientes que recebem orientações dos profissionais de saúde e são estimulados a cuidar do pé diabético adquirem melhores hábitos de autocuidado. Dessa forma, programas de educação baseados em diretrizes da prática clínica, tanto no ambiente das UBS como no ambiente hospitalar, devem ser estimulados e colocados em prática^16^. As atividades educacionais podem ser realizadas por meio de medidas criativas e atrativas, com uso da linguagem verbal apropriada ao paciente, vídeos, desenhos animados, imagens descritivas e discussões em grupo^32^^,^^33^.

Além disso, as ações educativas devem ser dialógicas para estimular a participação ativa dos pacientes e contextualizadas com simulações para o treino e a realização adequada do autocuidado^33^. Essas atitudes são relevantes para reduzir complicações, pois estudos evidenciam que 49 a 85% das úlceras podem ser evitadas com a educação em saúde que estimula a adesão do paciente ao plano terapêutico e, consequentemente, ao autocuidado^16^^,^^22^.

A maioria dos entrevistados afirmou possuir o hábito de inspecionar os pés, medida importante para detectar sinais precoces de ulceração, como a presença de edema, eritema, ressecamento excessivo, descoloração e lesões, como calosidades, feridas, perfurações e cortes^32^^,^^34^.

Mais de 65% dos pacientes portadores de DM entrevistados hidratavam os pés, resultado compatível com outros estudos^15^^,^^34^. O ressecamento da pele é comum no DM em razão das alterações sudomotoras causadas pela neuropatia autonômica. Assim, a hidratação deve ser realizada três vezes ao dia e possui o objetivo de evitar a presença de pele ressecada, que constitui meio susceptível para o desenvolvimento de rachaduras e calosidades^35^. Muitos pacientes afirmaram já ter tido rachaduras e/ou calos. Essas lesões são pré-ulcerativas, facilitadoras da entrada de microrganismos e precursoras do surgimento de infecções e ulcerações^10^^,^^35^. As micoses também constituem porta de entrada para o surgimento de quadros infecciosos, principalmente de natureza polimicrobiana, sendo relatada a presença desse problema em cerca de 30% dos pacientes avaliados^34^^,^^35^. Uma maneira para evitar o surgimento das micoses é o hábito de secar os espaços interdigitais após a lavagem dos pés^34^, prática realizada por 54,5% dos pacientes.

Quase 82% dos entrevistados relataram não ter o hábito de andar descalço, resultado semelhante a outros estudos^36^, sendo fator essencial no autocuidado para evitar traumas externos imperceptíveis e elevados estresses mecânicos em portadores de DM. Em relação ao corte das unhas, 92% dos indivíduos avaliados afirmaram ter cuidado ao cortá-las, mas não foi questionado o modo de corte, que deve ser reto, com os cantos das unhas lixados e redondos para prevenir o desenvolvimento de lesões e unhas encravadas^33^.

Aproximadamente 91% dos pacientes não utilizavam sapatos adequados para o DM, atitude encontrada em outros estudos, em que mais de 90% das pessoas diabéticas também tinham esse hábito. O calçado ideal não apresenta costura no interior, é macio, é regulável com cadarços ou velcro, proporciona proteção total dos dedos, o solado pode ter até 3 centímetros, preserva os pés de traumas mecânicos, é adequado ao tamanho do pé e distribui as áreas de pressão^10^^,^^15^.

O uso de sapatos adequados é uma orientação que o profissional de saúde deve fornecer aos pacientes, pois reduz os riscos de desenvolvimento do primeiro quadro de úlceras, como também diminui a recorrência de ulceração em pessoas com alteração da pressão plantar decorrente de cicatrizes ulcerativas^13^^,^^32^. A não adesão ao uso dos sapatos terapêuticos pode estar correlacionada com o alto valor desses calçados, podendo impossibilitar a aquisição em razão da baixa renda de muitos pacientes. Outra questão refere-se à estética, pois vários indivíduos consideram os calçados feios e dão preferência a sapatos comuns, que ocasionam lesões^22^.

Evidenciou-se que 48,9% dos entrevistados não procurariam a UBS na presença de lesões nos pés antes de realizarem procedimentos caseiros, medida distante do autocuidado, pois os pacientes devem ser orientados a procurarem um profissional de saúde na presença de alterações e/ou lesões^10^ antes de realizarem práticas caseiras que podem ocasionar infecções e ulcerações no pé. Exemplos de métodos inadequados são o aquecimento dos pés com ferro e bolsa de água quente, bem como uso de ferramentas inapropriadas para cortar calos e tratar rachaduras^8^.

A escolaridade dos portadores de DM influencia positivamente na capacidade de realização do autocuidado com os pés^37^. Espera-se que os indivíduos com graus de estudo mais avançados apresentem maior compreensão de leituras sobre a doença e o seu tratamento, bem como das informações fornecidas pelos profissionais de saúde, e, assim, tenham comportamentos preventivos adequados^8^^,^^16^^,^^37^. Isso coincide com algumas medidas de autocuidado expostas, em que 75% dos analfabetos não inspecionam os pés diariamente, e 58,3% desse mesmo grupo não procuram a UBS em casos de lesões e alterações nos pés. Nesse estudo, houve diferença estatística nas questões referentes ao costume de andar descalço, hidratar os pés, cortar as unhas, usar calçados adequados e identificar micoses, apresentando associação dessas medidas de autocuidado com a baixa escolaridade dos portadores de DM. Os profissionais de saúde devem produzir ações educacionais particularizadas para ajudar essa população, com orientações expostas de forma simples e objetiva, com linguagem compatível ao seu grau de escolaridade para, desse modo, proporcionar o conhecimento e estimular a realização do autocuidado^8^^,^^37^.

Quanto às respostas sobre a realização do autocuidado de acordo com o sexo, não houve diferença estatística no nosso estudo. Foi visto que cerca de 54,5% das mulheres tinham o hábito de hidratar os pés, enquanto apenas 11,4% dos homens exerciam esse hábito. Outras práticas encontradas que são mais realizadas pelo sexo feminino referem-se ao maior cuidado em secar os pés após lavagem e ao corte correto das unhas; já os homens possuíam maior porcentagem no hábito de uso dos sapatos adequados para o DM, resultados compatíveis com um estudo realizado na região Sul do Brasil^36^. O maior uso de sapatos adequados pelo sexo masculino relaciona-se a grande variedade de calçados utilizados pela população feminina, com saltos altos, aberturas e maior exposição dos dedos^38^. Desse modo, é importante que os serviços de saúde considerem as diferenças de gênero para construção de abordagens educativas e planos de cuidado mais eficientes^38^.

A úlcera do pé diabético é uma das complicações mais prevalentes em longo prazo, e cerca de 20% dos pacientes que vivem com DM têm alto risco de desenvolverem essa complicação nos pés em razão da presença de neuropatia^12^^,^^39^. A existência de úlcera é uma das principais causas de hospitalizações, sendo de difícil tratamento, com cerca de 40% de úlceras que não curam com medidas específicas nos 6 primeiros meses^12^.

Assim, as complicações do pé diabético correlacionam-se com baixa produtividade, custos individuais com a saúde, dificuldades para praticar exercícios físicos e problemas relacionados a ansiedade, depressão e estresse, bem como aqueles que intensificam sentimentos de isolamento social. Essas condições contribuem para a redução na qualidade de vida dos pacientes portadores de DM^12^.

É importante destacar as limitações presentes neste estudo. O número total de pacientes portadores de DM avaliados no estudo pode ter sido fator limitante por não ter alcançado o tamanho amostral calculado (considerando-se a prevalência do diagnóstico médico de DM de 7,4% no estudo do VIGITEL 2019, a população de Paulo Afonso de 118.516 habitantes, a margem aceitável de erro de 5% e o nível de significância de 95%, resultando em um tamanho amostral de 105 indivíduos). No entanto, essa amostra ainda permite inferências e extrapolação dos resultados, pois é representativa da população com DM. Também não foi quantificado o número de pessoas que se recusaram a participar da pesquisa e que foram excluídas; contudo, essas limitações não interferem na importância do estudo na medida em que esse tema é fundamental para a organização de ações educativas nos serviços de saúde.

## CONCLUSÃO

Evidenciou-se que os portadores de DM entrevistados não realizaram todas as medidas consideradas de autocuidado com os pés, como inspeção diária, hidratação, corte das unhas, secagem dos espaços interdigitais e uso de calçado adequado, além de desconhecerem o termo “pé diabético”. Houve associação entre menor escolaridade e menor capacidade de realização de medidas de autocuidado com os pés; não houve, porém, relação entre essas medidas de autocuidado e o sexo.

Nesse contexto, infere-se que a educação e o letramento em saúde, realizados e/ou reforçados pelos profissionais de saúde da APS de forma adaptada a cada perfil de paciente, são importantes para a melhoria do autocuidado com os pés e podem contribuir para a diminuição de complicações, hospitalizações e amputações nos pacientes portadores de DM.
